# The soluble form of CD160 acts as a tumor mediator of immune escape in melanoma

**DOI:** 10.1007/s00262-022-03199-0

**Published:** 2022-04-15

**Authors:** Marie-Léa Gauci, Jérôme Giustiniani, Clémence Lepelletier, Christian Garbar, Nicolas Thonnart, Nicolas Dumaz, Arnaud Foussat, Céleste Lebbé, Armand Bensussan, Anne Marie-Cardine

**Affiliations:** 1 INSERM U976, HIPI, Team 1 “Onco-Dermatology and Therapies”, Saint Louis Hospital, 1 avenue Claude Vellefaux, 75010 Paris, France; 2 Université Paris Cité, IRSL, Paris, France; 3 Institute Godinot, Reims, France; 4 Department of Dermatology, AP-HP, Saint-Louis Hospital, Paris, France; 5 Alderaan Biotechnology, Paris, France

**Keywords:** CD160, Melanoma, Tumor micro-environment, Immuno-suppression

## Abstract

Melanoma is responsible for 90% of skin cancer-related deaths. Major therapeutic advances have led to a considerable improvement in the prognosis of patients, with the development of targeted therapies (BRAF or MEK inhibitors) and immunotherapy (anti-CTLA-4 or -PD-1 antibodies). However, the tumor constitutes an immunosuppressive microenvironment that prevents the therapeutic efficacy and/or promotes the development of secondary resistances. CD160 is an activating NK-cell receptor initially described as delineating the NK and CD8^+^ *T*-cell cytotoxic populations. Three forms of CD160 have been described: (1) the GPI isoform, constitutively expressed and involved in the initiation of NK-cells' cytotoxic activity, (2) the transmembrane isoform, neo-synthesized upon cell activation, allowing the amplification of NK cells' cytotoxic functions and (3) the soluble form, generated after cleavage of the GPI isoform, which presents an immuno-suppressive activity. By performing immunohistochemistry analyses, we observed a strong expression of CD160 at the primary cutaneous tumor site of melanoma patients. We further demonstrated that melanoma cells express CD160-GPI isoform and constitutively release the soluble form (sCD160) into the tumor environment. sCD160 was shown to inhibit the cytotoxic activity of NK-cells towards their target cells. In addition, it was found in the serum of melanoma patients and associated with increased tumor dissemination. Altogether these results support a role for sCD160 in the mechanisms leading to the inhibition of anti-tumor response and immune surveillance in melanoma.

**Supplementary Information:**

The online version contains supplementary material available at 10.1007/s00262-022-03199-0.

## Introduction

Melanoma is one of the most aggressive skin cancers with a poor prognosis and an increasing frequency worldwide. Despite new treatment strategies that revolutionized melanoma management and outcomes (e.g. immunotherapy with anti-CTLA-4 or anti-PD-1 antibodies and/or targeted therapy with BRAF or MEK inhibitors), the 5-years survival of melanoma patients remains comprised between 20 and 60% depending on the treatment [1–5]. Improvement in advanced melanoma outcomes would therefore rely on the development of new therapeutic tools among which the one targeting molecules involved in the inhibition of anti-tumor immune responses. The tumor microenvironment consists of a highly complex and dynamic network of cells, soluble factors, signaling molecules (as the negative co-stimulatory receptors CTLA-4 and PD-1), extracellular matrix elements (e.g. cancer associated fibroblasts) and mechanical cues dedicated to promote tumor immune escape [6]. The latter is mainly characterized by decreased immunogenicity and cellular anti-tumor responses, a phenomenon known as immunoediting [7, 8]. It is essentially characterized by a shift of immune responses from a Th1 to a Th2 phenotype and an enhancement of immunosuppressive signals that ultimately support tumor growth and invasion, shield the tumor from the host immunity and foster therapeutic resistance [9].

NK lymphocytes are the main cellular actors of innate immunity which function is dedicated to the elimination of abnormal cells such as virally-infected or malignant cells. Their cytotoxic activity is governed by a fine-tuned balance between inhibitory and activating signals that tilts from one side to the other according to the nature of the ligands encountered on the target cells. A superiority of activating receptors' dependent pathways over inhibitory receptors signaling is mandatory for the development of an NK cell-mediated cytotoxicity. Nonetheless, recognition of HLA molecules by MHC class I NK receptors represents one of the most decisive interaction that dictates target cells' sparing or killing according to the self/missing-self theory.

CD160 is a NK cell receptor initially identified as an activating receptor expressed as an homodimer on peripheral blood NK and CD8^+^ T lymphocytes carrying cytotoxic activity [10]. It is encoded by a gene located on human chromosome 1, contains a single Ig-like domain and is linked to the plasma membrane through a glycosyl phosphatidyl inositol (GPI) anchor [11]. Its identified ligands are classical and non-classical MHC class I molecules and herpes virus-mediated entry (HVEM) protein [12, 13]. Consequently, CD160-GPI was characterized as an activating receptor as its engagement induces early NK cell cytotoxicity through a PI3-kinase-dependent pathway, potentiates IFN-*γ*, TNF-*α*, IL-6 and IL-8 cytokine production and plays a role in the early control of tumor growth [13–16]. During the process of NK cell activation, CD160-GPI is cleaved and released as a soluble form (sCD160) that has the property to inhibit NK and CD8^+^ *T* cell-mediated cytotoxicity [17, 18]. It is thought that sCD160 inhibitory function relies on its ability to interact with HLA molecules and to therefore interfere with their ligation to activating MHC-class I receptors. Beside the GPI form, a transmembrane isoform was also characterized (CD160-TM) that results from an alternative splicing of *CD160* gene [19]. CD160-TM expression was initially found highly restricted to activated NK cells. Furthermore, CD160-TM was characterized as an activating receptor, its engagement potentiating activated NK cell cytotoxicity through an as yet not fully identified signaling pathway. However, unlike CD160-GPI, CD160-TM was characterized as able to interact with MHC-class I molecules but not with HVEM [20].

CD160-GPI and CD160-TM representing specific markers for resting and activated NK lymphocytes respectively, we thought to use CD160 expression for the detection of NK cell infiltrates within a panel of solid tumors. Strikingly, we observed a strong tumor-associated positive signal in primary biopsies from melanoma patients. This observation prompted us to further investigate CD160 potential function in this peculiar tumor context.

## Material and methods

### Patients and cells

Blood and skin samples were obtained from melanoma patients after informed and written consent (ethic committee authorization No 2019-A02465-52). Patients’ main characteristics are described in supplementary Table 1.

Human melanoma cell lines (MEWO, COLO829, WM1361 and SKMel5) and the human chronic myelogenous leukemia cell line K562 were cultured in RPMI-1640 or DMEM medium supplemented with penicillin (100 IU/ml), streptomycin (100µL/ml), L-glutamine (2 mM) and 10% heat inactivated fetal calf serum (Life Technologies). Human melanocytes were maintained in Medium 254 supplemented with penicillin (100 IU/ml) and 1% human melanocytes growth supplement (Life Technologies). Peripheral blood mononuclear cells (PBMC) were isolated from heparinized venous blood by density gradient centrifugation over LSM (EuroBio).

## Anti-CD160 antibodies

H3 and H4 monoclonal antibodies (mAb; muIgG1) were generated by immunization of mice with a recombinant dimeric sCD160 protein. Recognition of an His-tagged sCD160 fusion protein by ELISA was used as criterion of selection. H3 mAb was further characterized as able to recognize both CD160-GPI and CD160-TM under denaturing conditions (e.g. immunofluorescence or Western blot analysis). A12 antibody (huIgG1) is a fully humanized antibody obtained by phage display and selected for its specific reactivity with CD160-TM, but not CD160-GPI, -expressing cells by flow cytometry. Conversely, BY55 mAb (muIgM) is a commercially available mAb (R&D Systems) that allows detection of cell membrane-associated CD160-GPI, but not CD160-TM, by flow cytometry. Antibodies specificities are shown in Supplementary Fig. 1.

## RNA extraction, reverse transcription and PCR

Total RNA was isolated using an extraction kit (RNeasy Plus Mini Kit, Qiagen) and reverse transcription was performed with the GoScript Reverse Transcriptase kit according to the manufacturer's recommendation (Promega). PCR was performed using Emerald PCR Master mix (Takara) and couples of primers allowing the specific detection of CD160-GPI or CD160-TM full length and $\Delta$Ig isoforms’ cDNA, as previously described [19]. The amplified products were separated on a 1% agarose gel and visualized with a FastGene Blue/Green LED GelPic Imaging System.

## Generation of CD160-negative cell lines

WM1361 cells were transfected with control or CD160 Mission shRNA plasmid DNA (hpgK-puro-CMV-tGFP, TRCN0000057579 or TRCN0000414945; Merck/Sigma-Aldrich) and JeTPei transfection reagent according to the manufacturer's protocol (Polyplus transfection). Transduced cells were selected by addition of 2.5 $\mu$g/ml puromycin to the culture medium, and GFP positive cells further sorted by flow cytometry leading to a cell purity over 99% (Supplementary Fig. 2).

## Immunohistochemistry

Formalin-fixed paraffin-embedded skin biopsies were labeled as previously described [18]. Briefly, 4 $\mu$M thick sections were subjected to antigen retrieval by heating in citrate buffer. Slides were then incubated with H3 mAb (10 $\mu$g/ml) followed by a secondary antibody and peroxidase, and detection with diaminobenzidine.

## Immunoprecipitation and Western blot

For total cell lysates analysis, cells were washed in PBS and resuspended in lysis buffer (1% Triton X100, 1 mM PMSF, 1 mM sodium vanadate, 20 mM Tris–HCl ph7.5, 10 mM NaF, 150 mM NaCl, 1 $\mu$g/ml aprotinin and 1 $\mu$g/ml leupeptin) for 1 h at 4 °C. Detection of sCD160 was performed on cell culture supernatants as previously described [17, 18]. Briefly supernatants were collected when cell confluence was reached, cleared by centrifugation for 15 min at 13,000 rpm and 4 °C, and subjected to a pre-clearing step using protein A-coupled Sepharose beads (GE Healthcare Biosciences) for 2 h at 4 °C. Immunoprecipitation was performed using H3 mAb or isotype-matched control muIgG1 and protein G-coupled Sepharose beads (GE Healthcare Biosciences).

After protein separation by SDS-10% PAGE under reducing (cell lysates) or non-reducing (immunoprecipitates) condition and transfer onto a nitrocellulose membrane, CD160 detection was performed using HRP-coupled H3 mAb and a chemiluminescent substrate (Perbio Sciences). Images were acquired with an Image Quant LAS4000 system (GE Healthcare Biosciences).

## Immunofluorescence

After adhesion on glass slides, melanoma cell lines were fixed in PBS/4% paraformaldehyde and permeabilized using PBS/0.5% Triton X100. After a blocking step in PBS/3% goat serum/3% BSA, anti-CD160 (H3) and anti-PD-L1 antibodies were added. After washes, a mix of AlexaFluor 488-coupled anti-mouse and AlexaFluor 594-coupled anti-rabbit Igs antibodies (Invitrogen) was added. Slides were mounted with a DAPI Fluoromount solution (Southern Biotech).

## Flow cytometry

Cells were labeled with PE- or APC-conjugated anti-PD-L1, anti-CD160 (clone BY55), anti-HVEM or anti-HLA-A/B/C antibodies or their respective isotype-matched control antibodies. When indicated, cells were fixed and permeabilized prior to labeling according to the Cytofix/Cytoperm protocol (BD Biosciences).

For detection of sCD160 binding, cells were pre-incubated for 1 h at RT with a His-tagged sCD160 fusion protein (sCD160-His), melanoma cell culture supernatants or culture medium (negative control). Ligation of sCD160 to the cells was further detected using an APC-coupled anti-His mAb or anti-CD160 H3 mAb plus APC-conjugated secondary antibodies. Where indicated, an anti-HLA-A/B/C mAb (clone W6/32) was added to the cells before incubation with sCD160-His.

After acquisition on a Cytoflex cytometer (Beckman Coulter), data analysis was performed using FlowJo software (Tree Star Inc.).

## Cytotoxicity assay

Target cells were either left untreated (WM1361) or labeled with CFSE (K562) according to the manufacturer's instruction (Invitrogen). Target cells were subjected to a pre-incubation step with culture medium or cell culture supernatant obtained from the indicated melanoma cell line. PBMC from healthy donors were then added at effector to target (E/T) ratios of 5/1, 10/1, 20/1 or 40/1. After a 4.5 h incubation at 37 °C, targets' apoptosis was evaluated by addition of 7-AAD as previously described [21]. Cells were analyzed by flow cytometry and results expressed as percentage of 7-AAD-positive cells among target cells.

## sCD160 ELISA

96-well plates were coated with the anti-CD160 H4 mAb (1 $\mu$g/well) ON at 4 °C. After washes, a blocking step was performed using PBS/0.5% BSA/1% Tween20 as blocking buffer (200 $\mu$l/well; 1h30 at RT). Sera prepared from healthy donors (HD; *n* = 16) or melanoma patients (*n* = 16) blood were added (200 $\mu$l/well), as well as serial dilutions of His-tagged sCD160 fusion protein (ranging from 0 to 30 ng/ml) allowing generation of a standard curve. Standards and samples were incubated for 2 h at RT and sCD160 binding revealed using HRP-conjugated H3 mAb as detection antibody (0.2 $\mu$g/ml; 2 h at RT) and TMB substrate (100 $\mu$l/well; Sigma-Aldrich). Series of 3 washes in PBS/0.5% BSA/1% Tween20 were performed between each revelation steps. After addition of stop solution (100 $\mu$l/well; Sigma-Aldrich), absorbance was measured at 450 nm on a Multiscan FC spectrophotometer (Thermo Scientific). Results were expressed as mean ± SD of triplicates.

## Statistical analysis

For each experiment, results are expressed as means ± SD of 3 independent experiments or triplicates. Statistical analyses were performed using a Mann–Whitney *t* test with Prism GraphPad software. *p* < 0.05 was considered as significant.

## Results

### CD160 is highly expressed in primary tumors of melanoma patients

**Fig. 1:**
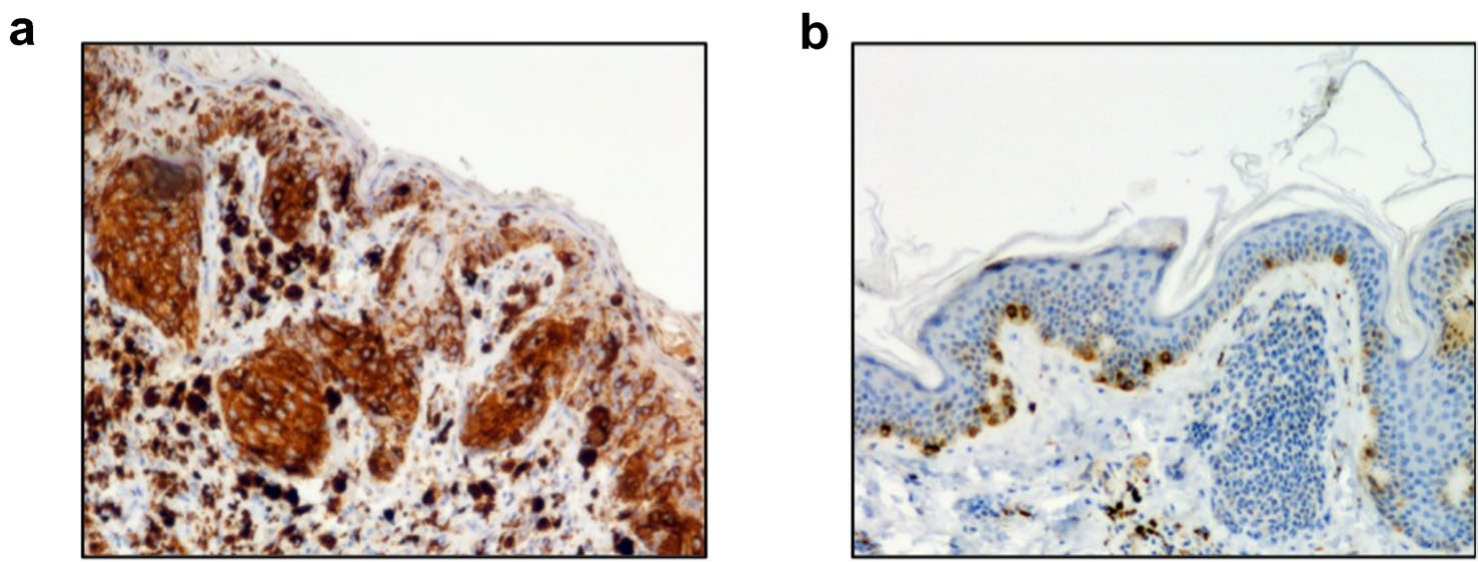
CD160 is expressed in melanoma primary cutaneous tumor. Single immuno-staining was performed using anti-CD160 mAb H3 on sections of lesional skin from **a** melanoma patients (*n* = 9) or **b** circumscribed nevi (*n* = 11). Shown are images from a representative sample from each group

Since our previous studies established that CD160 represents a reliable NK cell marker, we sought to evaluate the presence of NK cell infiltrates within solid tumors through the detection of CD160 expression. Biopsies from different tumor types were therefore analyzed by immunohistochemistry using the anti-CD160 mAb H3. Indeed, while presenting the inconveniency of recognizing both GPI and TM isoforms, H3 mAb was the only one reacting with paraffin-embedded cells in our preliminary tests (Supplementary Fig. 1b). Unexpectedly, a strong and positive signal was observed on cutaneous biopsies of primary tumor from melanoma patients (*n* = 9), that did not seem to delineate immune cells' infiltrates but rather the entire tumor tissue (representative biopsy labeling shown in Fig. 1a). Such positivity was not observed in biopsies obtained from benign human nevi (*n* = 11), where a discrete and cell-associated positive signal was detected (Fig. 1b). These first results prompted us to determine whether CD160 expression could be a characteristic of melanoma as well as its potential function in this skin tumor context.

## Melanoma cells express CD160-GPI but not CD160-TM

**Fig. 2:**
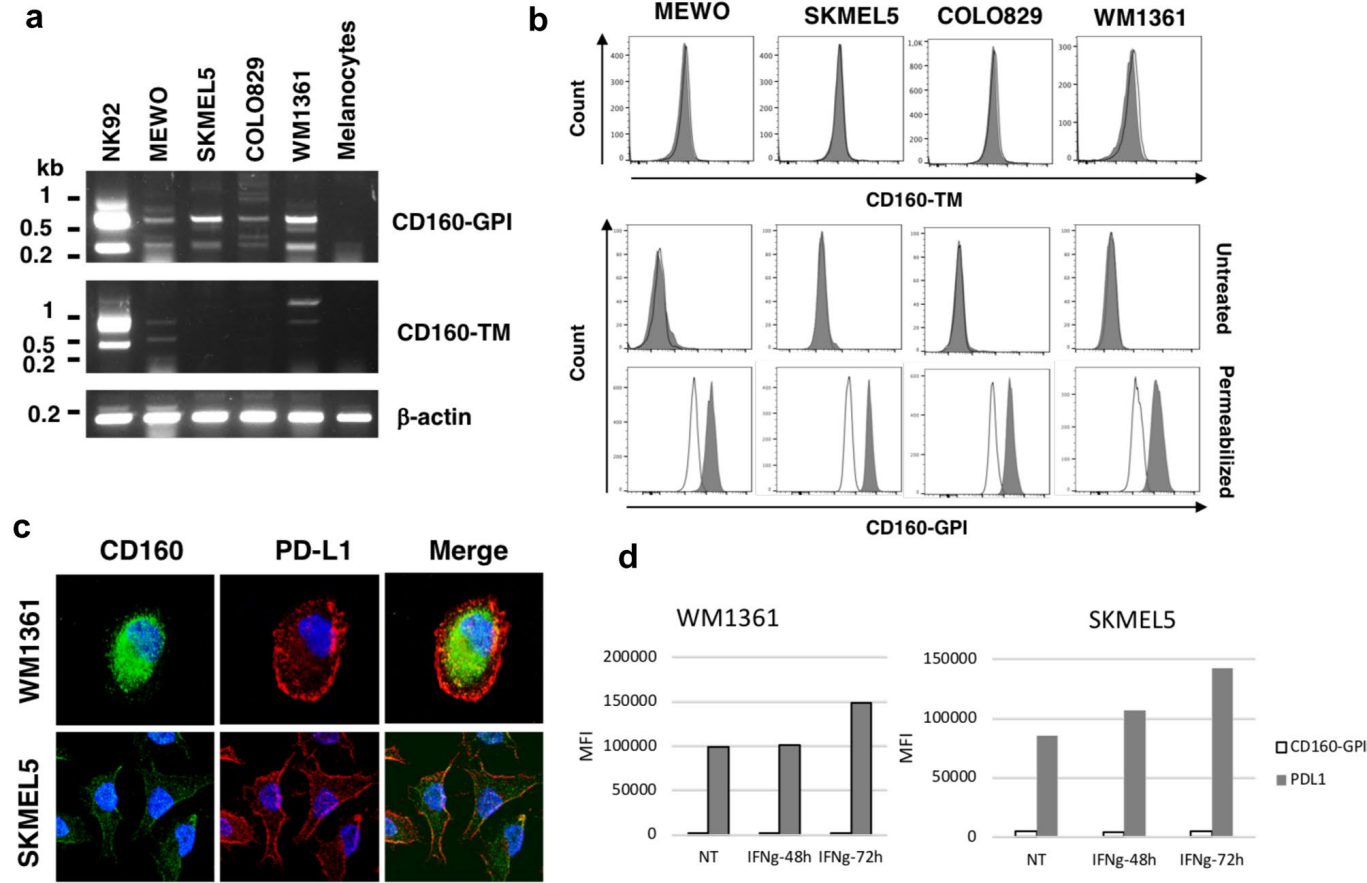
Expression of CD160-GPI, but not CD160-TM, by melanoma cells. **a** Total RNAs were extracted from melanoma cell lines (MEWO, COLO829, SKMEL5 and WM1361), melanocytes and NK92 cell line and subjected to reverse transcription. PCR were realized using a pair of primers corresponding to the 5′ and 3′ ends of CD160 or CD160-TM reported coding sequence. Amplification of *β*-actin cDNA was performed in parallel as an internal control. **b** Analysis of CD160-GPI and CD160-TM cell expression by human melanoma cells. Cells were labeled with APC-conjugated anti-CD160-TM Ab A12 (top panels) or anti-CD160-GPI mAb BY55 (middle and bottom panels; grey histograms) or their respective isotype control (white histograms). When indicated, cells were subjected to a fixation/permeabilization step to allow intracellular staining (bottom panels). Cells were then analyzed by flow cytometry. **c** Intracellular localization of CD160-GPI in melanoma cells. After adhesion on glass slides, fixation and permeabilization, SKMEL5 and WM1361 cells were incubated with the anti-CD160 mAb H3 or rabbit anti-PD-L1 antibody. After washes, a mix of AlexaFluor 488-coupled anti-mouse and AlexaFluor 594-coupled anti-rabbit Igs antibodies was added to allow detection of CD160 (green) and PD-L1 (red). Slides were then mounted with a Dapi-containing medium for nuclei visualization (blue). Images were acquired on a Zeiss LSM780 confocal microscope. Magnification: 60x. **d** Cells were left untreated (NT) or incubated with IFN$\gamma$ for 48 or 72 h. At each time point, cells were subjected to anti-CD160-GPI and anti-PD-L1 staining and analyzed by flow cytometry. Results are expressed as means ± SD of the mean fluorescence intensity (MFI) corresponding to CD160-GPI (white) and PD-L1 (grey) proteins observed in 3 independent experiments

To establish if CD160 expression could be directly achieved by melanoma cells, RT-PCR was first performed on RNA extracted from human melanoma cell lines (namely MEWO, COLO829, SKMEL5 and WM1361) and from their normal cellular counterpart, human melanocytes (HMN). Total RNA from the NK cell line NK92, previously shown to constitutively express CD160-GPI and CD160-TM transcripts, was used as positive control [19]. Two transcripts of 550 and 220 bp, corresponding to the cDNA encoding full length and $\Delta$Ig isoform of CD160-GPI, respectively, were found in all melanoma cell lines tested but not in normal melanocytes (Fig. 2a). In contrast, full length (700 bp) or $\Delta$Ig (380 bp) CD160-TM encoding transcripts were detected in MEWO and WM1361cell lines but were absent in SKMEL5, COLO829 and melanocytes (Fig. 2a).

We next aimed to determine if the synthesis of CD160 transcripts by melanoma cells was correlated with efficient protein expression. Flow cytometry analysis was performed using the anti-CD160-GPI mAb BY55 or the anti-CD160-TM Ab A12 (see antibodies specificity in Supplementary Fig. 1c). As shown in Fig. 2b, no expression of CD160-TM (top panels) or CD160-GPI (middle panels) was detected at the cell surface of melanoma cell lines. However, a positive signal was observed with the anti-CD160-GPI BY55 mAb following cell permeabilization (bottom panels), suggesting that CD160-GPI might be sequestered at the intracellular level in melanoma cells.

The cellular localization of CD160-GPI was further assessed by immunofluorescence using H3 mAb. Labelings were realized on the PD-L1 positive cell lines SKMEL5 and WM1361 to allow visualization of plasma membrane-associated signals through PD-L1 labeling. The obtained results confirmed that, while PD-L1 presented plasma membrane-associated and intracellular localizations, CD160 was only found within the cytoplasm with the detection of a diffuse labeling (Fig. 2c). In order to determine if CD160-GPI processing to the cell plasma membrane might be induced following activation, SKMEL5 and WM1361 cells were incubated in the presence of IFN$\gamma$, a cytokine previously described as enhancing PD-L1 expression on melanoma cells [22, 23]. Incubation of both cell lines with IFN$\gamma$ did not result in CD160-GPI membrane expression while an increased in PDL-1 membrane expression was detected (Fig. 2d).

## Melanoma cells release the soluble form of CD160 (sCD160) which presents binding ability to tumor cells

**Fig. 3:**
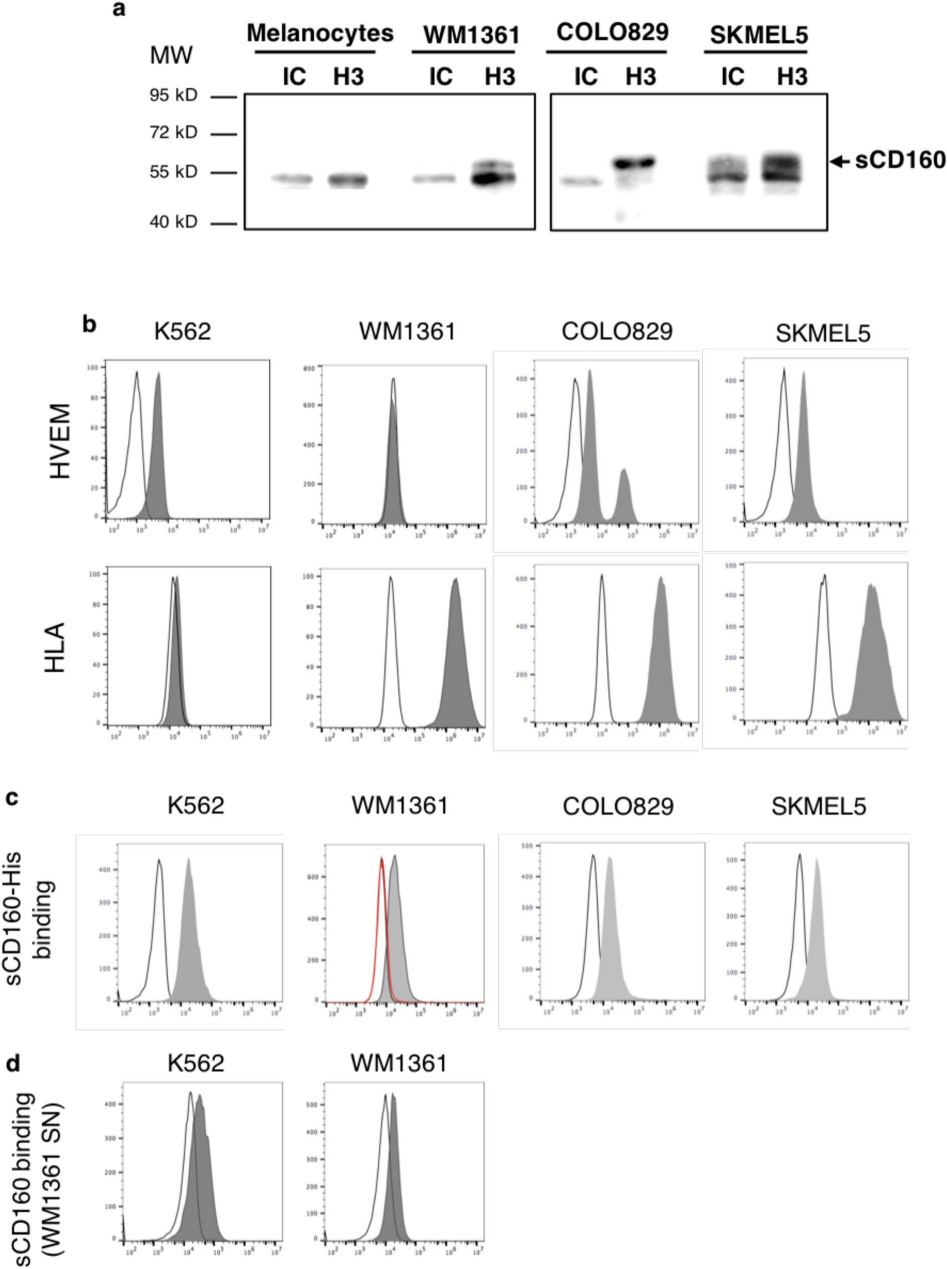
sCD160 is released by and binds to melanoma cells. **a** Culture supernatant (SN) was recovered from confluent culture of WM1361, COLO829 and SKMEL5 cells or melanocytes. cleared by centrifugation and subjected to immunoprecipitation using H3 mAb or isotype control murine IgG1 (IC). After separation by SDS-PAGE and transfer onto nitrocellulose, protein revelation was performed by incubation of the blot with HRP-coupled H3 mAb. **b** Expression of sCD160 ligands by K562 and melanoma cell lines. Cells were labeled with PE-conjugated anti-HLA-A/B/C or anti-HVEM mAb (grey histograms) or isotype control mAb (white) and analyzed by flow cytometry. **c** Binding of sCD160 to K562 and melanoma cell lines. Cells were pre-incubated with a His-tagged sCD160 fusion protein (sCD160-His) for 1 h at room temperature. After washes, ligation of sC160 was detected by staining with an APC-coupled anti-His mAb and flow cytometry analysis (grey histograms). Negative controls were performed in parallel (white histograms). An additional condition, where WM1361 cells were simultaneously incubated with sCD160-His and an anti-HLA-A/B/C mAb was also performed (red histogram). **d** Secreted sCD160 efficiently binds to K562 and WM1361 cell lines. Cells were pre-incubated with culture medium (negative control) or WM1361 culture supernatant for 1 h at room temperature. Ligation of sCD160 was detected with H3 anti-CD160 mAb plus APC-conjugated secondary antibodies

Our above data suggested that CD160-GPI is expressed by melanoma cells but not processed as a membrane-associated receptor in these cells. Interestingly, similar observations (CD160-GPI cell expression with no detection of the receptor at the cell membrane) were previously made on the human mast cell HMC-1 cell line [18]. However, this cell line was described as able to constitutively secrete CD160 under its soluble form. To test whether the same process might apply for melanoma cells, culture supernatants obtained after confluent growth (96 h-post-seeding) of human melanocytes or COLO829, SKMEL5 and WM1361 cell lines were collected and subjected to immunoprecipitation and immunoblotting with the anti-CD160 H3 mAb. As shown in Fig. 3a, a 55-kDa molecule, corresponding to the expected apparent molecular weight for dimeric sCD160, was specifically recovered from melanoma cell lines but not from normal melanocytes culture supernatant. Our data therefore established that melanoma cells produced CD160-GPI and directly released its soluble form into the extracellular environment.

We previously demonstrated that MHC-class I restricted cytotoxicity of CD8^+^ T cells or NK cells could be impaired in the presence of sCD160 [17, 24]. We therefore tested sCD160 ability to bind to K562 and melanoma cell lines. Flow cytometry analysis allowed detection of differentially expressed MHC-class I molecules and HVEM depending on the cell line. Thus, K562 showed an HLA-A/B/C^−^ HVEM^+^ phenotype while WM1361 were identified as HLA-A/B/C^+^ HVEM^−^ cells (Fig. 3b). In contrast, COLO829 and SKMEL5 showed a double positive HLA-A/B/C^+^ HVEM^+^ phenotype. Consequently, efficient binding of sCD160 to K562 and melanoma cells was observed when using a His-tagged sCD160 fusion protein (Fig. 3c). Finally, in agreement with its HLA^+^HVEM^−^ phenotype, sCD160 binding to WM1361 cell line was completely abrogated in the presence of a blocking anti-HLA-A/B/C mAb (Fig. 3c, red histogram). Unfortunately, our attempts to inhibit sCD160/HVEM interaction on K562 by using commercially available anti-HVEM antibodies remained unsuccessful. Because K562 and WM1361 were the only cell lines which binding to sCD160 relied on a unique ligand (HVEM or MHC-class I molecules, respectively), they were selected for further use as target cells in subsequent cytotoxicity assays (see below), once their ability to bind sCD160 produced by WM1361 has been verified (Fig. 3d).

## sCD160 secreted by melanoma cells inhibits NK cell-dependent cytotoxicity

To further assess if sCD160 released by melanoma cells could inhibit NK cell-dependent cytotoxic activity, allogeneic cytotoxic assays were conducted using PBMC as a source of NK cells (effector cells) and either K562 or WM1361 cell line as target cells.

**Fig. 4:**
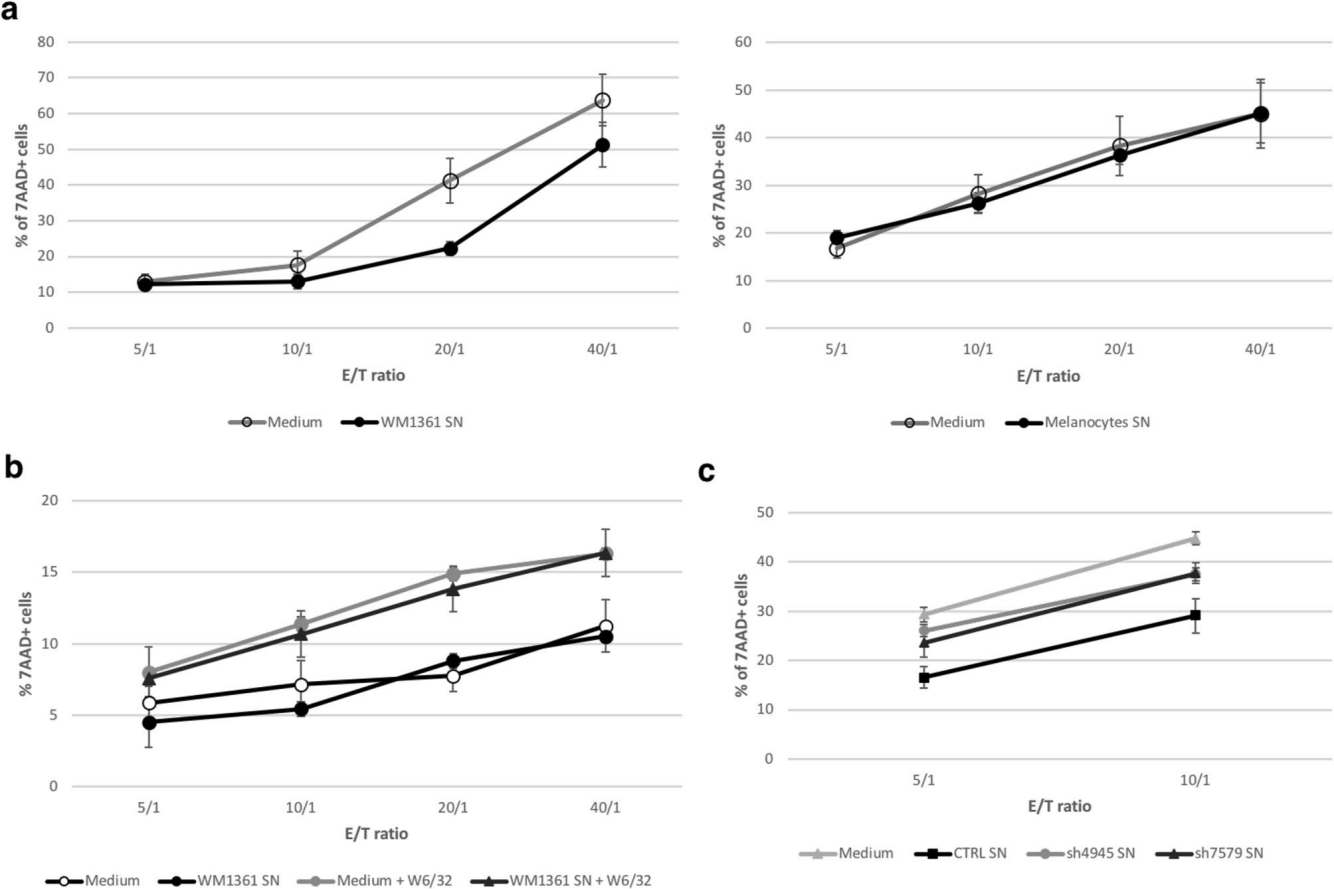
Inhibition of NK-cell dependent cytotoxicity by sCD160 released by melanoma cells. **a** K562 target cells were pre-incubated with WM1361 (left) or melanocytes (right) culture supernatant or their corresponding culture medium. PBMC from healthy volunteers were then added at the indicated E/T ratios. After incubation, target cells' death was monitored by 7AAD labeling and flow cytometry analysis. **b** WM1361 were either left untreated or pre-incubated with W6/32 anti-HLA-A/B/C antibody prior to contact with culture medium or WM1361 cell culture supernatant. PBMC were then added and targets' depletion monitored as described in (a). **c** Control (CTRL) or CD160-depleted (sh4945 and sh7579) WM1361 cells, expressing GFP alone or GFP plus CD160 shRNA, respectively, were obtained as described in the Material and Methods section. Culture supernatants were prepared from each cell type and use for pre-incubation of K562 target cells. Cytotoxic assays were then performed as described in (a). (a-c) Shown results corresponded to the mean ± SD of three independent experiments. * *p* < 0.05, ** *p* < 0.01, **** *p* < 0.001

The inhibition potential of secreted sCD160 on the natural cytotoxic activity of NK cells towards K562 cell line was first tested. An efficient killing of the target cells was detected when pre-incubated with fresh culture medium, while K562 pre-incubation with WM1361 culture supernatant led to a significantly reduced killing activity (Fig. 4a, left panel; *p* < 0.001 at *E*/*T* = 20/1). Such inhibition was not detected when using culture supernatant from melanocytes for K562 pre-incubation step (Fig. 4a, right panel). Similar experiments were performed using WM1361 cells as target cells but remained inconclusive. Indeed, due to their high expression level of MHC-class I molecules, no cytotoxic activity was developed by the effector cells against WM1361, the development of a NK-dependent killing activity requiring their pre-incubation with a blocking anti-HLA-A/B/C mAb (Fig. 4b). Since we previously demonstrated that sCD160 binding to WM1361 cells was abrogated in the presence of the anti-HLA mAb (Fig. 3c), sCD160 showed no effect on the cytotoxic activity developed in these conditions.

To therefore provide direct evidence that the inhibitory potential of WM1361 cell supernatant was based on the presence of secreted sCD160, CD160-negative WM1361 cell lines were generated by transfection with a plasmid allowing both green fluorescent protein (GFP) expression and CD160 shRNA synthesis. To ascertain the specificity of CD160 RNA depletion, plasmids encoding for two distinct RNA sequences were used for *CD160* gene knockdown (referred to as sh4945 and sh7579 thereafter). A control plasmid, encoding for GFP alone, allowed generation of a control CD160^+^ GFP^+^ cell line (CTRL). After selection and GFP expression-based cell sorting by flow cytometry, pure populations were obtained for each cell type, on which CD160 protein expression was further assessed by Western blot (Supplementary Fig. 2). Culture supernatants were prepared from control and CD160-depleted cells and further used in the pre-incubation step of K562 target cells in our allogeneic cytotoxicity assay. As demonstrated in Fig. 4c incubation of K562 cells with CTRL cells supernatant resulted in a strong inhibition of the NK cell-dependent cytotoxicity, as previously observed with the parental cell line. By comparison, a significantly enhanced depletion of the target cells was observed when supernatants prepared from CD160-depleted cells were used. Taken together our results demonstrated that sCD160 secreted by melanoma cells mediates inhibition of NK cell-dependent cytotoxicity.

## Detection of sCD160 in the serum of melanoma patients

**Fig. 5:**
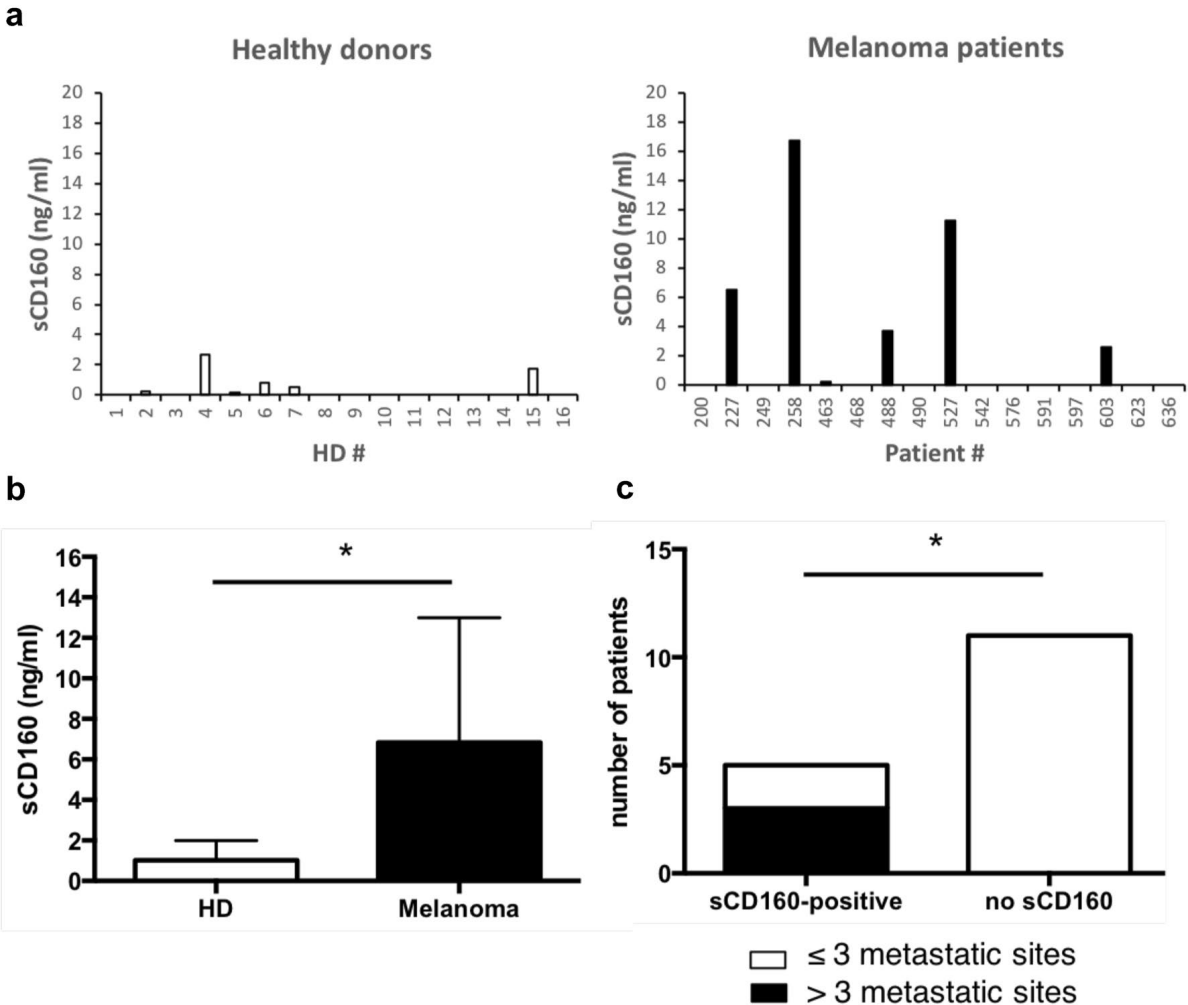
The presence of sCD160 in melanoma patients' blood is correlated with high number of metastatic sites. **a** sCD160 dosage was performed by ELISA on the serum obtained from healthy blood donors (left; *n* = 16) or melanoma patients (right; *n* = 16). Shown are the mean ± SD of triplicates. **b** Mean ± SD of sCD160 concentrations detected among the positive HD or melanoma patients’ samples (*n* = 6 in each group). Statistical analysis was performed using a Mann Whitney *t* test. * *p* < 0.05. **c** Contingency analysis of sCD160 dosage and the number of metastatic sites detected for each melanoma patient. Statistical analysis was performed using a Fisher's exact test. * *p* < 0.05

While NK cells were found under-represented in the bulk of tumor-infiltrating lymphocytes, they represent 5 to 15% of peripheral blood lymphocytes. It has been therefore suggested that, if not directly involved in the mechanisms promoting tumor depletion, they might play a crucial role in the surveillance and elimination of circulating tumor cells to avoid their dissemination and the occurrence of metastases [25, 26]. One could then hypothesize that, if present in the blood stream, sCD160 would be deleterious for NK lymphocytes' sentinel function. To further test this hypothesis, we developed an anti-sCD160 ELISA test for evaluating the presence of sCD160 in the serum of melanoma patients. Detection was also performed on similar samples obtained from healthy volunteers as control. As shown in Fig. 5a, sCD160 was detected in 6 out of 16 (37.5%) tested samples in both HD and melanoma patients' groups. However, the concentrations of sCD160 found in melanoma patients' sera were significantly higher than in HD sera (median = 0.67 ng/ml and range: 0.2–2.66 ng/ml for HD; median = 5.105 ng/ml and range: 0.21–16.74 ng/ml for melanoma patients; *p* < 0.05; Fig. 5b). Flow cytometry analysis, performed on the same cohorts of HD and melanoma patients, did not reveal any decrease in the percentage of circulating CD160-GPI positive NK cells neither between the two groups nor for the 6 melanoma patients exhibiting high levels of sCD160 (data not shown), suggesting that the presence of sCD160 in the blood flow did not result from membrane-bound CD160-GPI proteolytic cleavage. Finally, in light with sCD160 potential role in hampering NK cells' immune surveillance function, crossed analyses of seric sCD160 dosages and the number of metastatic sites in melanoma patients was performed. Notably, all patients presenting more than 3 metastatic sites (*n* = 3/16) showed high levels of circulating sCD160 (Fig. 5c). This latter observation therefore supports the idea that secretion of sCD160 by the tumor might be part of the process leading to tumor escape and dissemination.

## Discussion

CD160-GPI was initially described as an activating membrane receptor expressed by cytotoxic NK and CD8^+^ T cells [11]. Indeed, we and others established that its engagement promotes NK cell cytotoxicity and is mandatory for IFN$\gamma$ production [13, 16]. Over the last years, CD160-GPI expression was further evidenced on other cell types including activated endothelial cells and human mast cells [18, 27]. Thus, CD160-GPI was found expressed on the endothelial cells of newly formed blood vessels in human colon carcinoma and in a mouse B16 melanoma model but not in vessels of healthy tissues [28]. Consequently, an anti-CD160 mAb with anti-angiogenic properties has been characterized, its injection promoting regression of the tumor vasculature and normalization of the remaining vessels in B16 melanoma-bearing mice, but also inhibition of ocular neovascularization in rabbit cornea or in a mouse model of oxygen-induced retinopathy [27, 28]. Beside endothelial cells, CD160-GPI expression by human mast cells was also investigated. A release of CD160 soluble form (sCD160) by mast cells in an inflammatory context has been reported, as well as its ability to inhibit MHC-class I-dependent CD8^+^ T cell cytotoxicity [18].

In the present study, we brought novel insights into the function of sCD160 as a tumor factor that could be involved in the inhibitory mechanisms of anti-tumor immune responses. We established that melanoma cells expressed CD160-GPI and secreted sCD160 in their extracellular environment. Moreover, this expression appeared constitutive and independent of the tumor mutational status, as it was observed in all cell lines tested that presented the most common melanoma-associated mutations, namely BRAF/V600E, NRAS/Q61K and NF1. In NK cells, sCD160 release requires prior cellular activation and results from the proteolytic cleavage of membrane-bound CD160-GPI receptors. Our data suggest that, in melanoma cells, the process of sCD160 production is constitutive and occurs through a classical protein secretion pathway as it remained exclusively detected at the cytoplasmic level but not at the cell surface, even after cell treatments known to induce expression of membrane-associated proteins such as PD-L1 (e.g. INF-$\gamma$ [22]).

Over the last years, several mechanisms conditioning tumor immune escape were highlighted including, among others, the release of immunosuppressive cytokines, molecules, or soluble factors by the tumor. This tumor-associated microenvironment was shown to promote the recruitment of immunosuppressive cells such as regulatory *T* cells, tumor-associated macrophages or myeloid-derived suppressor cells, the deregulation of endothelial cells' growth and adhesion pathways, or the inhibition of cytotoxic T cells function, all processes built to impair *T* cells recruitment or killing ability [29, 30]. Impairment of NK cell-dependent immunosurveillance and anti-tumor activity has also been reported. The so far described mechanisms included the down-regulation of activating (e.g. NKG2D) and up-regulation of inhibitory (Tim-3, LAG3, PD-1, …) receptors or conversely, the shedding or down-expression of activating ligands (ULPB, MICA/B) and over-expression of inhibitory molecules (PD-L1, non MHC-class I ligands) by the tumor cells [31, 32]. We here described that the constitutive release of sCD160 by melanoma cells might be part of the inhibition processes leading to ineffective NK cell-dependent anti-tumor depletion. Indeed, we demonstrated that sCD160, released by melanoma cell lines, is able to bind to HLA molecules or HVEM on target cells and consequently to promote inhibition of NK cell-dependent cytotoxic activity. In addition, sCD160 was found in the blood stream of 6 out of 16 melanoma patients tested, among which 3 were the one presenting more than 3 metastatic sites at the time of blood collection. While these data have to be confirmed on a larger cohort of patients, and retrospectively confronted to parameters such as patients' overall response or survival, they support the idea that sCD160, once produced by the tumor, actively participates to NK cell sentinel function silencing.

## Supplementary Information

Below is the link to the electronic supplementary material.

Supplementary file1 (PDF 590 KB)
