# Supplementary material for: The soluble form of CD160 acts as a tumor mediator of immune escape in melanoma
Source: Cancer Immunol Immunother. 2022 Apr 15;71(11):2731–42. doi: 10.1007/s00262-022-03199-0 (PMC9519731; doi:10.1007/s00262-022-03199-0)
Supplement: Supplementary file 1 — Supplementary file1 (PDF 590 KB) [file 262_2022_3199_MOESM1_ESM.pdf]

SUPPLEMENTARY FIGURES

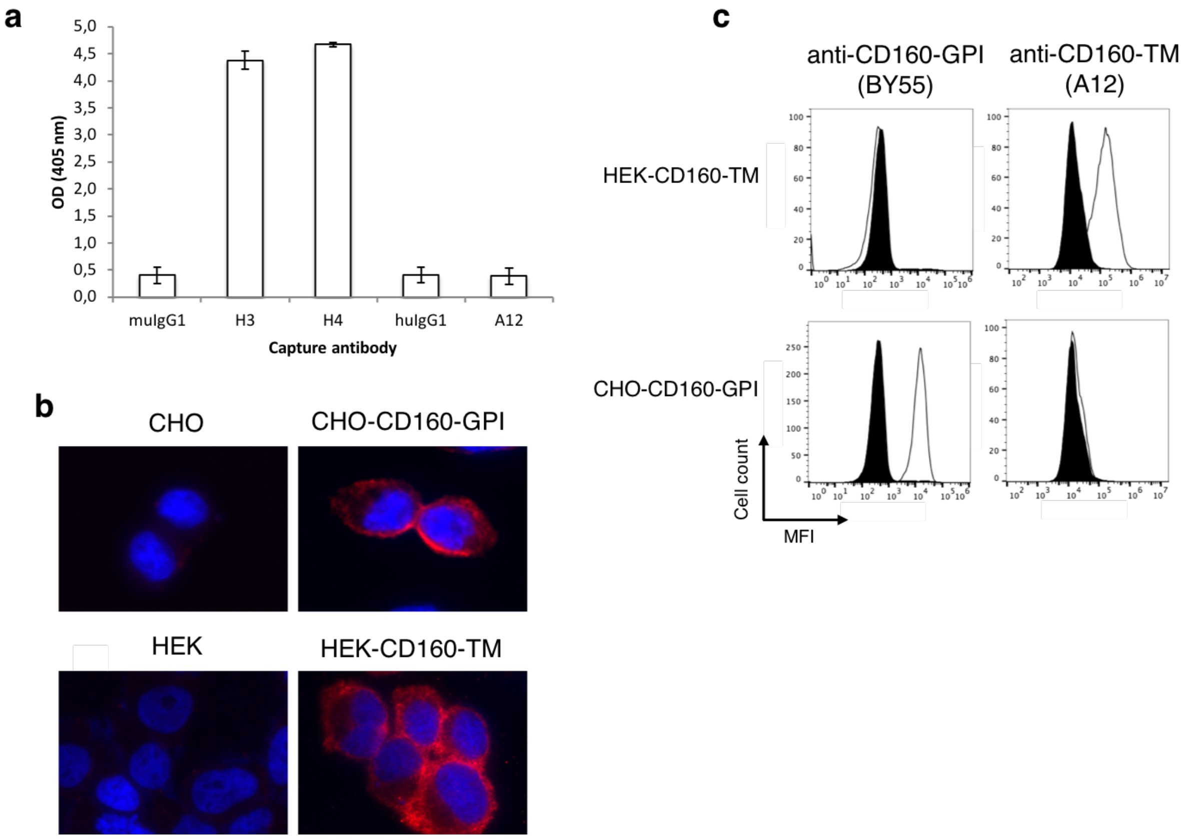

Supplementary Fig. 1: Specificity of the antibodies used in this study. (a) H3, H4 and huA12 specificity towards soluble CD160 (sCD160) was assessed by ELISA. Each antibody (or its respective isotype control) was used as capture antibody. After incubation with an His-tagged sCD160 fusion protein, revelation was performed using an HRP-conjugated anti-His mAb and TMB substrate. **(b)** Immuno-fluorescence labeling was performed with H3 mAb on wild type or stably transfected CHO or HEK cell lines forced to express either CD160-GPI (CHO) or CD160-TM (HEK) isoform. **(c)** Immuno-staining was performed on CHO-CD160-GPI or HEK-CD160-TM cells using APC-conjugated BY55 or huA12 antibody. Cells were further analyzed by flow cytometry.

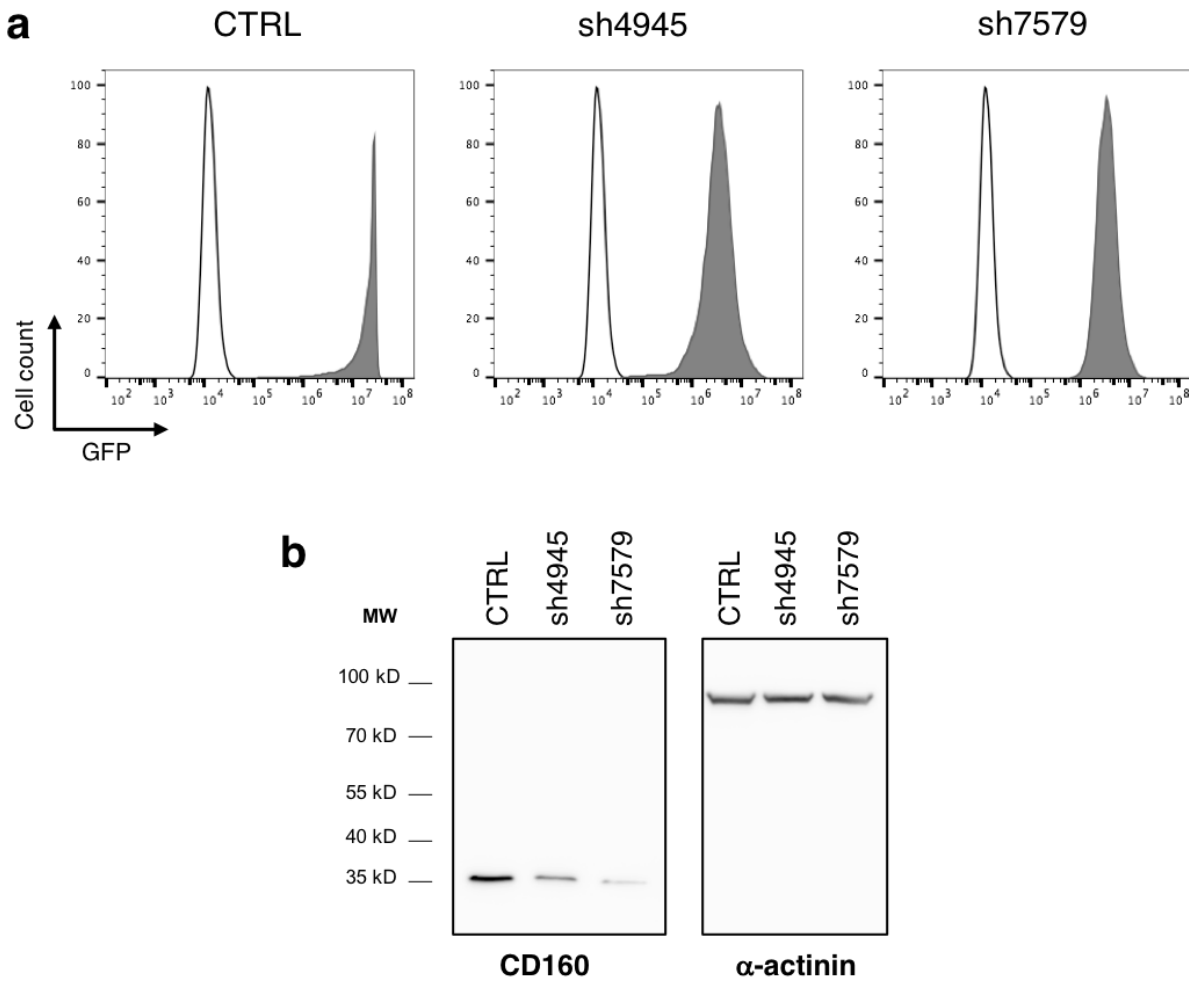

Supplementary Fig. 2: Generation of CD160-depleted WM1361 cell lines. (a) After transfection with a control plasmid (CTRL) or shCD160-containing plasmid (sh4945 or sh7579) and antibiotic selection, cells were subjected to cell sorting, amplified and analyzed by flow cytometry for their GFP positivity. **(b)** Decreased expression of CD160 was assessed by Western blot using H3 mAb. Equal protein loading was verified through detection of α-actinin.

SUPPLEMENTARY TABLE:

Supplementary Table 1: Patients main features (n=16)

Gender (M/F)	11/5
Age (years)	Mean: 59.25 / Range: 31-81
Stage (n)	III (6), IV (10)
Mutational status (n)	WT (7), B-RAF (9)
Number of metastatic sites (n)	Mean: 2.3 / Range: 1-5
Treatment at inclusion (n)	Immunotherapy:<br>- Ipilimumab + Nivolumab (10)<br>- Nivolumab (3)<br>Targeted therapy (3)<br>Combo (1)
Time of treatment (months)	Mean: 9,75 / Range: 0-55
Response status to treatment (n)	PD (3), PR (10), CR (1), NA (2)

PD: progressive disease; PR: partial response; CR: complete response; NA: not applicable.
